# Are Effect Sizes in Emotional Intelligence Field Declining? A Meta-Meta Analysis

**DOI:** 10.3389/fpsyg.2019.01655

**Published:** 2019-07-16

**Authors:** Zhun Gong, Xinian Jiao

**Affiliations:** Department of Psychology, Normal College, Qingdao University, Qingdao, China

**Keywords:** emotional intelligence, decline effects, ability emotional intelligence, mixed emotional intelligence, replicability crisis

## Abstract

Since Salovey and Mayer (1990) first proposed the emotional intelligence (EI) as an independent intellectual component, research on the field of EI has developed rapidly. A large number of studies have shown that emotional intelligence is an important predictor that affects our lives, such as job performance, mental health, and so on. However, we observed that some effect sizes in the field of emotional intelligence decreased over time. Is this kind of decline simply due to random errors, or is emotional intelligence field undergoing decline effects? The present study analyzed 484 effect sizes based on the responses of 102,579 participants from nine meta-analyses in emotional intelligence field to estimate the average effect size, and evidence for decline effects in this field. This study finds that the average effect size of EI is 0.244 (*p* < 0.001), and the average effect size of mixed EI (*r* = 0.272, *p* < 0.001) is significantly higher than that of ability EI (*r* = 0.160, *p* < 0.001). Effect sizes in the field of EI decrease with time, there are decline effects in emotional intelligence field. Furthermore, there are also decline effects in mixed EI field. However, we find no evidence that there are decline effects in ability EI field. Base on the significant average effect size of mixed EI, the most likely explanation for the decline in effect sizes is that effect sizes of mixed EI in the original studies were overestimate. This study considers decline effects in mixed EI research as inflated decline effects. To sum up, decline effects in the field of emotional intelligence are mainly due to the choice of emotional intelligence model and measurement method.

## Introduction

Can results of psychological research be replicated? With the discovery that a large number of published psychological research results could not be successfully replicated in new samples, the problem has received increasing attention, and researchers have found that many results were overly optimistic and effect sizes were overestimated (Francis, 2014). Based on this, COS organization repeated 100 psychological studies (Hartgerink and Pernet, 2015), they found that the average effect size in repetitive studies was half of that in the original studies. The original studies' results in 97% were statistically significant, but only 36% of repetitive studies had significant results. Therefore, the researchers began to doubt the credibility of psychological research results and discuss “replicability crisis” (Baker, 2016). The replicability crisis is a term used by psychologists in the current introspection phase, and the psychology community is undergoing a revolution (Spellman, 2015).

Because of the increasing emphasis on the role of emotional intelligence in today's society, it is generally accepted that the predictive power of emotional intelligence measurement to individual success transcends the predictive power of traditional cognitive measurement (Zeidner et al., 1998). Therefore, since the first systematic study of emotional intelligence by Mayer and Salovey in the 1990s, emotional intelligence has quickly aroused widespread concern and scientific interest among many scholars. Research on emotional intelligence has been in full swing for the past 30 years. So, does emotional intelligence field also experience a replicability crisis?

The decline effect is the phenomenon of decreasing effect sizes in repeated studies over time (Schooler, 2011). Decline effect is an important indicator to explore the replicability crisis. Therefore, the present study examines whether emotional intelligence experiences a replicability crisis by studying decline effects in the field of emotional intelligence.

### Emotional Intelligence

Psychologists give many different definitions of emotional intelligence, represented by three genres, which are more popular in psychology:

The first genre is ability model. They defined emotional intelligence as “the ability to perceive accurately, appraise, and express emotion; the ability to access and/or generate feelings when they facilitate thought; the ability to understand emotion and emotional knowledge; and the ability to regulate emotions to promote emotional and intellectual growth” (Mayer and Salovey, 1997). They separated personality traits from emotional intelligence through a multi-factor intelligence scale and claimed that emotional intelligence was independent of personality traits (Mayer et al., 2003).

The second genre is Goleman's emotional intelligence model. Unlike Salovey and Mayer's views, Goleman considered that emotional intelligence is based on both cognitive ability and personality trait. He defined emotional intelligence as a universal ability that every normal person has, and a quantifiable dimension that embodies individual differences, and that individuals can be sorted by emotional measurements within a certain range (Goleman, 1995). Moreover, the four important components that make up emotional intelligence were: self-awareness, social awareness, self-management and interpersonal management (Goleman, 1996). In 1998, Goleman improved his model and proposed the concept of emotional competence, which he defined as “an ability to recognize, understand and use emotional information about oneself or others that leads to or causes effective or superior performance” (Goleman, 1998).

The third genre is Bar-On model. Bar-On defined emotional intelligence as “a cross-section of interrelated emotional and social competencies, skills and facilitators that determine how effectively we understand and express ourselves, understand others and relate with them, and cope with daily demands.” (Bar-On, 1997). And there were the five key components of emotional intelligence included: Intrapersonal, Interpersonal, Stress Management, Adaptability and General Mood (Bar-On, 2004). Based on these five components, he designed the first standardized emotional intelligence scale (Bar-On Emotion Quotient Inventory, EQ-i) (Bar-On, 2006).

These three genres can be divided into two models, namely, mixed model and ability model. Mayer and Salovey's ability model belongs to ability EI, whereas, Goleman's model and Bar-On model belong to mixed EI. Mixed EI and ability EI are completely different conceptual frameworks, rather than different means of measuring the same concept. Ability model considers emotional intelligence to be an independent intellectual component and uses objective measurement methods. Whereas mixed model considers that emotional intelligence is distinct from personality and general cognitive ability, and uses the self-report scale (Bar-On, 2000; Byrne et al., 2007). Based on differences between two models, the present study will be discussed separately decline effects in mixed EI and ability EI.

Since Salovey and Mayer (1990) first proposed EI as an independent intellectual component, research on the field of EI has developed rapidly. Many studies have shown that EI is not only an important factor affecting individual success, but also a direct factor affecting individual mental health (Stewart-Brown, 1998; Bar-On, 2016).

Many studies carried out in recent years have linked EI with a number of variables. some studies have reported that individuals with high EI were more likely to have strong social adaptability and more emotional skills, thus individuals with high EI were more likely to report positive relationships with others and more parental support (Fabio, 2015). Some studies showed that EI influenced life satisfaction (Extremera and Rey, 2016). Some studies have also found that EI could positively predict job performance and academic performance (Bar-On et al., 2006; Newman et al., 2010; Sharma, 2011; Bar-On, 2018). And EI could negatively predict drug and alcohol involvement (Peterson et al., 2011). In addition, EI was negatively correlated with suicidal behavior (Domínguez-García and Fernández-Berrocal, 2018). To sum up, EI is an important topic in psychological research, and the research on EI is developing rapidly (Sharma, 2008).

### Decline Effect

The decline effect is a phenomenon in which effect sizes decrease with time, and it is a pattern of bias. Protzko and Schooler (2015) divided decline effects into 4 types: False positive decline effects, Inflated decline effects, Under-specified decline effects and Genuinely decreasing decline effects.

False positive decline effects mean that effect sizes of subsequent studies decrease over time, because there is no true effect. Significant results in the original studies originated from the errors of statistics or methods. For example, Mozart's music has non-significant effect on cognition, the original result was a statistical fluke (Pietschnig et al., 2010).

Inflated decline effects mean that effect sizes of subsequent studies decrease over time, because original studies overestimated effect sizes (Protzko and Schooler, 2015). For instance, effect sizes that men with symmetrical secondary sexual characteristics had advantage in selecting mates decreased, because for reducing measurement errors subsequent studies no longer used single-exposure methods (Swaddle et al., 1994).

Under-specified decline effects mean that effect sizes of subsequent studies decrease over time, because a necessary condition in original studies was under-specified. For example, in the economic game of intuition promoting cooperation, the original study did not report that all the participates were new to this game, which led to a decline in effect sizes of subsequent studies (Rand et al., 2012).

Genuinely decreasing decline effects mean that effect sizes of subsequent studies decrease over time, due to social developments. For instance, with the cultural development, the prejudice of white students toward African Americans decreased (Dovidio and Gaertner, 1986).

Therefore, are there decline effects in emotional intelligence field? If so, what type of decline effects are they?

### Meta-Meta Analysis

Meta-Meta analysis is a quantitative re-analysis on a series of meta-analyses, and it is an important way to examine whether there are biases in a certain field (Cumming, 2012, 2014; Fox et al., 2014). The rationales of Meta-Meta analysis are the same as that of meta-analysis, and there are also strict screening criteria and coding system. However, there are still differences, for example, the research object of meta-analysis is primary study, and the research object of Meta-Meta analysis is meta-analysis. Additionally, Meta-analysis is mainly applicable to study relationship between variables or whether interventions are effective, whereas Meta-Meta analysis is mainly used to study biases in a certain field. Biases affect the development of science, and they are important causes of replicability crises. Therefore, Metascience could rescue replicability crises (Schooler, 2014).

Fanelli and Ioannidis (2013) used Meta-Meta analysis to re-analyze 82 meta-analyses in softer research, and found evidences of “US effect.” US effect means that “US studies may overestimate effect sizes in softer research” (Fanelli and Ioannidis, 2013). Then, Fanelli et al. (2017) using Meta-Meta analysis to explore biases in the whole field of science (including Small-study effects, Gray literature bias, Decline effect, Early-extreme, Citation bias, US effect and Industry bias). Following Fanelli et al. (2017), we will look at decline effects in the field of emotional intelligence.

### Objectives and Hypotheses

By reading the literature, we find that predictive effects of emotional intelligence seem to be declining. For example, the predictive effect of mixed EI on academic performance has decreased over time. In 1998, Schutte reported the effect size (*r*) between mixed EI and academic performance was 0.374 (Schutte et al., 1998). In 2005, Austin reported the effect size (*r*) between those was 0.283 (Austin et al., 2005). And in 2010, Radford reported the effect size (*r*) of that was 0.112 (Radford, 2012).

Effect sizes of the ability EI field are also observed decreasing. In 2006, Côté and Miners reported the effect size (*r*) between ability EI and cognitive ability was 0.47 (Côté and Miners, 2006). Then, in 2011, Fiori and Autonakis reported the effect size (*r*) between those was 0.36 (Fiori and Antonakis, 2011). In 2014, Grunes reported the effect size of that was 0.26 (Grunes et al., 2014).

Effect sizes in the field of emotional intelligence appear to be declining. Is this kind of decline simply due to random errors, or is emotional intelligence field undergoing decline effects? All above lead to the present study with the following objectives (O) and hypotheses (H):

O1. Determine whether there are decline effects in the field of EI. We hypothesized that there were decline effects in the field of EI, in other words, effect sizes in the field of EI decreased with time (H1).

O2. Determine whether there are decline effects in the field of mixed EI. We hypothesized that there were decline effects in the field of mixed EI, in other words, effect sizes in the field of mixed EI decreased with time (H2).

O3. Determine whether there are decline effects in the field of ability EI. We hypothesized that there were decline effects in the field of ability EI, in other words, effect sizes in the field of ability EI decreased with time (H3).

O4. Discuss the specific type of decline effects in the field of EI. And explore the causes of decline effects in the field of EI.

## Methods

### Search Strategy and Selection Criteria

We searched for meta-analyses about EQ and emotional intelligence on the 28th of September, 2018, on ISI Web of Knowledge, using the search string “TOPIC: (EQ OR Emotional Intelligence) AND TOPIC: (meta-analysis).” This rendered 258 records. And the present study selection criteria included that meta-analysis should be unique, available, quantitative, and related to emotional intelligence, using at least one specific emotional intelligence measure and providing full data table.

**Figure 1:**
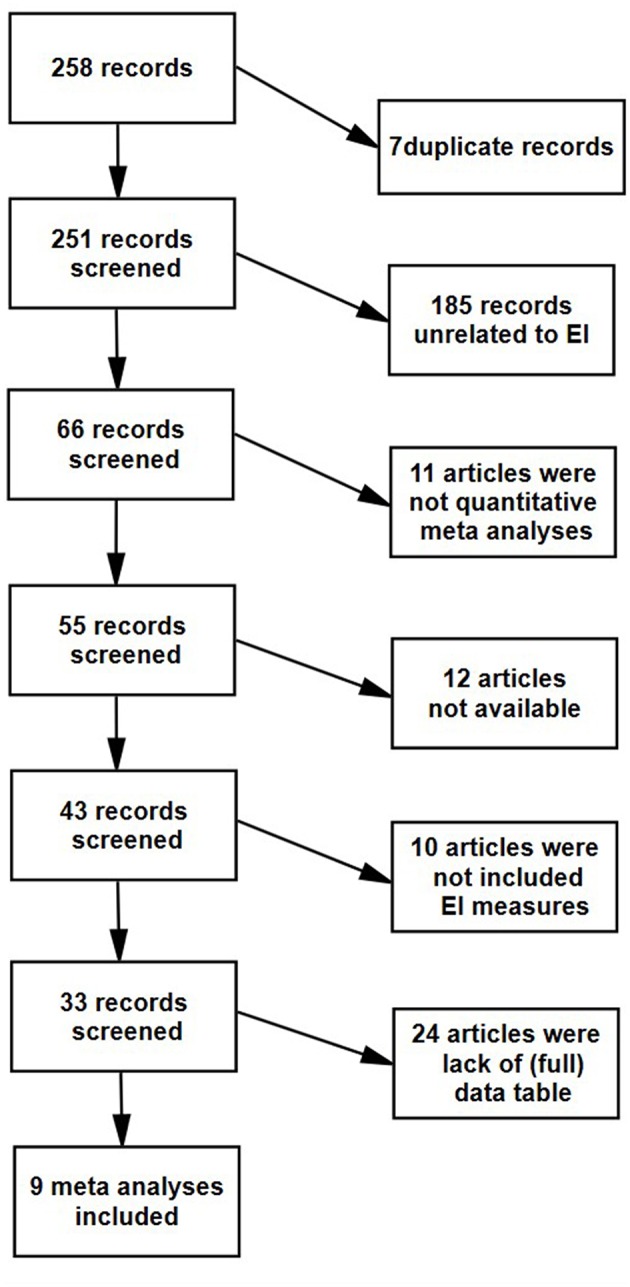
A schematic overview of the exclusion criteria and meta-analysis selection.

From these 258 records, we excluded seven duplicate ones and 185 records unrelated to emotional intelligence. We then excluded 11 articles that were not a quantitative meta-analysis, and 12 records in which the article was not available in our university library. We excluded 10 articles, because articles need to include at least one specific emotional intelligence measure (EI measures see Table 1). We then excluded 24 articles because of lack of (full) data table, non-standard method, or too few studies. For one primary study, we were not able to calculate the effect size, because information on effect size was missing, so this was excluded from our analyses. Our final sample consisted of nine meta-analyses, included 484 unique primary studies, and based on 102,579 participants (see Figure 1 for a schematic overview of the exclusion criteria and meta-analysis selection).

**Table 1:** Emotional intelligence measure.

**EI model**	**Task**	**Full name of task**
Ability EI	MSCEIT	Mayer, Salovey, Caruso Emotional Intelligence Test (Mayer et al., 2002)
	MEIS	Multifactor Emotional Intelligence Scale
Mixed EI	EIQ	Emotional Intelligence Questionnaire (Dulewicz et al., 2003)
	EISRS	Emotional Intelligence Self-Regulation Scale (Martinez-Pons, 1999–2000)
	EQ-i	Emotional Quotient Inventory (Bar-On, 1997)
	SEIS	Schutte Emotional Intelligence Scale (Schutte et al., 1998)
	TEIQ	Traits Emotional Intelligence Questionnaire (Tsaousis and Nikolaou, 2005)
	TEIQue	Trait Emotional Intelligence Questionnaire (Petrides et al., 2007)
	TMMS	Trait Meta Mood Scale (Salovey et al., 1995)
	WLEIS	Wong and Law Emotional Intelligence Scale (Law et al., 2004)
	ECI	Emotional Competence Inventory (Sala, 2002)
	AES	The Assessing Emotions Scale (Schutte et al., 2009)
	EII	Emotional Intelligence Inventory (Tapia, 2001)
	BRIEF	Brain Resource Inventory for Emotional intelligence Factors (Kemp et al., 2005)

### Coding Procedures

After the literature screening, we encoded all the primary studies from collected meta-analyses, in which repetitive primary studies were coded only once. This way, we ensured the independence of the primary studies. For each unique primary study, we recorded the effect size and its standard error. Next, we recorded the primary studies' total sample size and the sample size per condition. Then, we recorded the publication year of the primary study, and we coded the relative order of publication year (studies published in different months of the same year are regarded as published at the same time). Furthermore, we coded the EI model which the primary study was based on. We created a binary variable to indicate if a study was based on mixed model (1) or ability model (0). To ensure reliability of the present study was robust, two independent recorders (i.e., the first author and the second author) double-coded all the primary studies from collected meta-analyses, achieving an intra-class correlation (ICC) of 0.95 of coding comparison, and then two independent recorders discussed and corrected discrepancies in individual coding.

### Effect Size Conversion

For meta-analyses, it should convert all the effect sizes to a single type. All the effect size we extracted from nine meta-analyses were correlation (*r*), so we chose correlation (*r*) as the type of effect size. We subsequently converted all *r*'s to Fisher's *Z* values, because the standard error then only depends on the sample size and not on the correlation itself (Sterne and Egger, 2006).

Direction of effect sizes can affect results. To correct for any influence of direction, we used a procedure called “coining,” following Fanelli et al. (2017). This procedure assumed that if the meta-analytic average effect size was negative, the expected direction of primary effect sizes was also negative. And it should multiply all primary effect sizes whose direction was negative by −1. In the present study, we considered the relationship between trait psychopathy and EI as negative, and we also considered the relationship between alcohol involvement and EI as negative.

## Results

All meta-analyses in the present study were carried out using CMA 2.0. Publication bias analysis, heterogeneity analysis, average effect size calculation, meta regression were carried out, and results were as follow.

### Publication Bias

**Figure 2:**
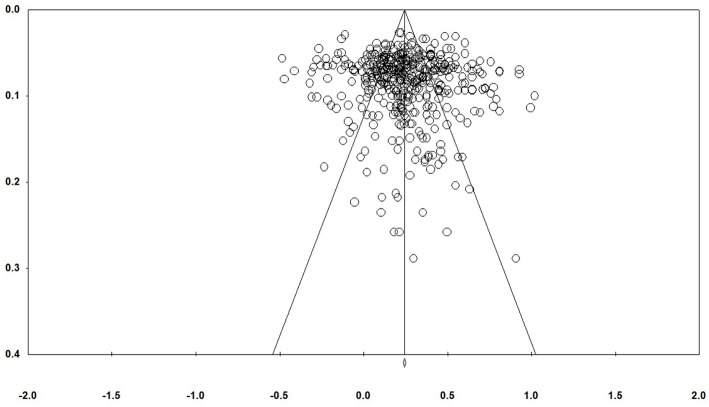
Funnel Plot of Standard Error by Fisher's Z.

The analysis of published bias involves study size and effect size. The mechanism for displaying the relationship between study size and effect size is the funnel plot. Traditionally, the funnel plot was plotted with effect size on the X axis and the sample size or variance on the Y axis. In the absence of publication bias, the studies will be distributed symmetrically about the mean effect size, since the sampling error is random. Figure 2 shows that there is little likelihood of publication bias in emotional intelligence field. Therefore, the results of the present study are not affected by publication bias.

### Heterogeneity Analysis

As shown in Table 2, over all *Q* statistic is 4803.808 and its *p*-value is less than 0.001, which shows that effect sizes in the field of EI are heterogeneous. And effect sizes in the field of mixed EI are heterogeneous (*Q* = 3882.462, df = 375, *p* < 0.001), effect sizes in the field of ability EI are also heterogeneous (*Q* = 734.104, df = 107, *p* < 0.001). And the difference between mixed EI and ability EI is significant (*Q* = 187.241, df = 1, *p* < 0.001). Therefore, we should choose random-effects model rather than fixed-effects model in following analyses.

**Table 2:** Heterogeneity analysis.

	***Q*-value**	**df(Q)**	***p*-value**	**I^**2**^**
Ability EI	734.104	107	<0.001	85.424
Mixed EI	3882.462	375	<0.001	90.341
Total between	187.241	1	<0.001	
Over all	4803.808	483	<0.001	89.945

### Average Effect Size Calculation

As shown in Table 3, the average effect size of EI is 0.244 (*p* < 0.001). The average effect size of mixed EI is 0.272 (*p* < 0.001), and the average effect size of ability EI is 0.160 (*p* < 0.001). The average effect size of mixed EI is significantly higher than that of ability EI.

**Table 3:** Average effect size calculation.

	**k**	***N***	**Effect size and 95% interval**			***Z*-value**	***P*-value**
			**Point estimate**	**Lower limit**	**Upper limit**		
Ability EI	108	19,313	0.160	0.122	0.197	8.111	<0.001
Mixed EI	376	83,266	0.272	0.251	0.293	24.089	<0.001
Over all	484	102,579	0.244	0.226	0.263	24.892	<0.001

### Meta Regression

**Figure 3:**
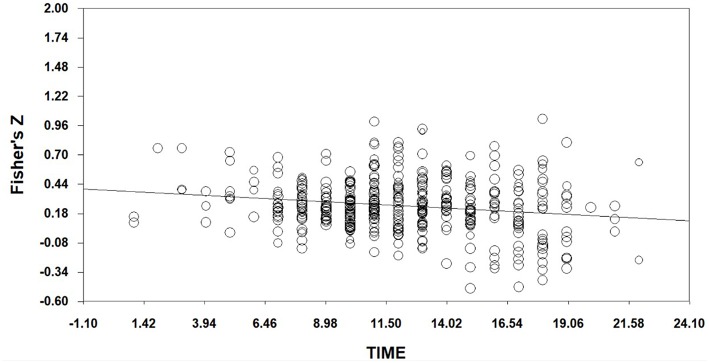
Regression of Time on Fisher's Z (EI).

As shown in Table 4, the relationship between effect sizes of overall EI and time is stronger than we would except by chance (*Q*_model_ = 17.09059, df = 1, *p* < 0.001). With time in the model, the between-studies variance can be explained (*Q*_residual_ = 480.98990, df = 482, *p* > 0.05). And the slope is less than zero significantly (*Z* = −4.13408, *p* < 0.001). Therefore, the relationship of time to effect is *y* = −0.01133x+ 0.38726, where x is the order of publication year. We can plot this in Figure 3. It illustrates that effect sizes in the field of EI decreases with time, there are decline effects in emotional intelligence field. This indicates that hypothesis 1 is acceptable.

**Table 4:** Mixed effects regression of time (EI).

	**Point estimate**	**Standard error**	**Lower limit**	**Upper limit**	***Z*-value**	***P*-value**
Slope	−0.01133	0.00274	−0.01670	−0.00596	−4.13408	<0.001
Intercept	0.38726	0.03393	0.32077	0.45376	11.41506	<0.001
	**Q**	**df**	***P*****-value**			
Model	17.09059	1	<0.001			
Residual	480.98990	482	0.51601			
Total	497.18049	483	0.31800			

**Figure 4:**
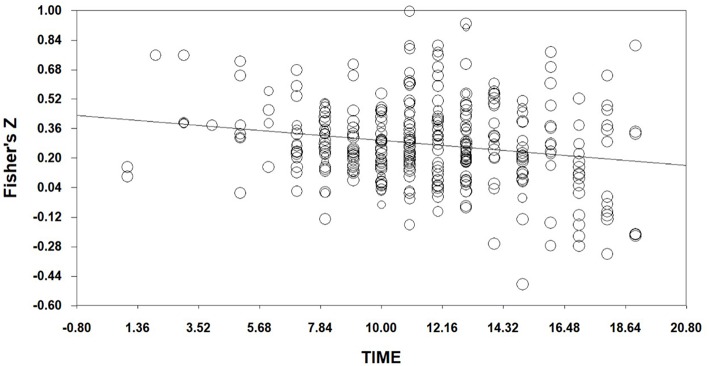
Regression of Time on Fisher's Z (Mixed EI).

**Figure 5:**
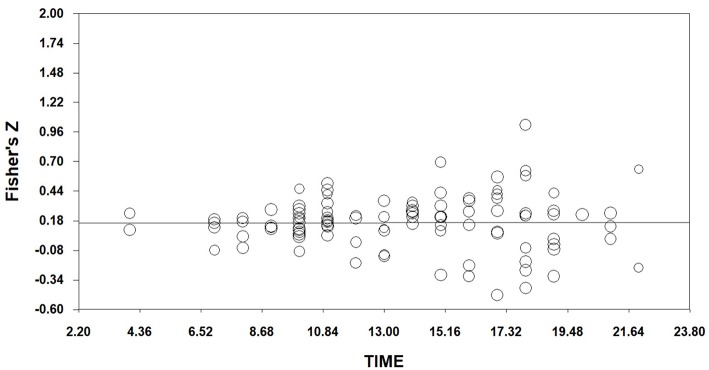
Regression of Time on Fisher's Z (Ability EI).

In Table 5, the relationship between effect sizes of mixed EI and time is significant (*Q*_model_ = 14.72518, df = 1, *p* < 0.001). With time in the model, the between-studies variance can be explained (*Q*_residual_ = 369.30960, df = 374, *p* > 0.05). And the slope is less than zero significantly (*Z* = −3.83734, *p* < 0.001). Therefore, the relationship of time to effect is *y* = −0.01247x+ 0.42108, where x is the order of publication year. We can plot this in Figure 4. It shows that effect sizes in the field of mixed EI decreases with time, there are decline effects in mixed EI field. This indicates that hypothesis 2 is acceptable. In Table 6, the relationship between effect sizes of ability EI and time is non-significant (*Q*_model_ = 0.00218, df = 1, *p* > 0.05). We can plot this in Figure 5. It illustrates that effect sizes in the field of ability EI do not decrease with time, we find no evidence that there are decline effects in ability EI field. This indicates that hypothesis 3 is rejected.

**Table 5:** Mixed effects regression of time (Mixed EI).

	**Point estimate**	**Standard error**	**Lower limit**	**Upper limit**	***Z*-value**	***P*-value**
Slope	−0.01247	0.00325	−0.01883	−0.00610	−3.83734	<0.001
Intercept	0.42108	0.03864	0.34536	0.49681	10.89846	<0.001
	**Q**	**df**	***P*****-value**			
Model	14.72518	1	<0.001			
Residual	369.30960	374	0.55877			
Total	384.03478	375	0.36256			

**Table 6:** Mixed effects regression of time (Ability EI).

	**Point estimate**	**Standard error**	**Lower limit**	**Upper limit**	***Z*-value**	***P*-value**
Slope	0.00024	0.00518	−0.00991	0.01039	0.04665	0.96279
Intercept	0.15848	0.07243	0.01652	0.30045	2.18806	0.02867
	**Q**	**df**	***P*****-value**			
Model	0.00218	1	0.96279			
Residual	110.40040	106	0.36545			
Total	110.40258	107	0.39137			

## Discussion

**Figure 6:**
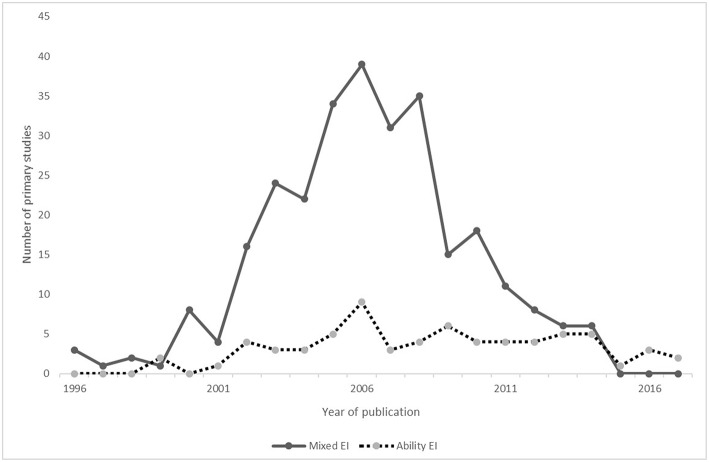
Line chart.

The present study is the first to our knowledge that provides evidences that there are decline effects in the field of EI. This finding is consistent with hypothesis H1 and with previous studies showing that psychology is facing replicability crises. There are two possible explanations for this. One is that effect sizes of mixed EI field decrease, because there are 77.7% primary studies based on the mixed model. The other is that the number of primary studies in mixed EI decrease sharply after 2008 (see Figure 6). Though the average effect size of mixed EI is significantly higher than that of ability EI, the number of primary studies in mixed EI decrease, leading to the decrease of effect sizes in entire EI. However, the latter explanation is limited by sample size of the present study, the total sample size before 2008 was twice as big as that after 2008, so the number of primary studies in mixed EI decrease, possibly due to smaller total sample size.

Our finding is also consistent with hypothesis H2, suggesting that effect sizes in mixed EI decline over time, which is the most important finding in this study. According to Protzko and Schooler's classification of decline effects (see section Decline Effect), there are 4 types of decline effects: False positive decline effects, Inflated decline effects, Under-specified decline effects and Genuinely decreasing decline effects. And here is a discussion about the type of decline effects in mixed EI. This kind of decline effects does not belong to false positive decline effects, because of the significant average effect size of mixed EI. Various effects in the entire mixed EI field decline rather than a particular effect, and there are no unclear specific conditions, therefore this kind of decline effect is not under-specified decline effect. The emphasis on emotional intelligence does not decline with society development, so this does not belong to genuinely decreasing decline effects. Base on the significant average effect size of mixed EI, the most likely explanation for this decline is that effect sizes of mixed EI in the original studies were overestimated. Therefore, we consider decline effects in mixed EI research as inflated decline effects.

And there are three possible reasons why effect sizes in mixed EI were overestimated. One is that mixed model does not have a high degree of independence. There is a high overlap between mixed EI and general factors of personality (Rooy and Viswesvaran, 2004; Linden et al., 2017; Alegre et al., 2019). And there are overlaps among mixed EI, optimism, and positive emotions (Davies et al., 1998). The second reason is the change of mixed EI measurement method. The original measurement method of mixed EI is self-report scale, and the reliability and objectivity of it has been controversial. Studies have found that the level of intelligence by self-report scale had nothing to do with its actual level of intelligence (Paulhus, 2010). Therefore, psychologists supplemented 360-degree feedback based on the original self-report scale. Three hundred and sixty degree Feedback refers to “different raters—typically a manager (boss), peers, direct reports, and others to rater a specific person” (Adrian et al., 2014). Three hundred and sixty degree feedback effectively improves the accuracy and reliability of measurements (Furnham, 2008). Psychologists are also trying experimental methods and neuroscience methods such as brain mapping (Takeuchi et al., 2013).

Thirdly, with deepening studies on emotional intelligence, “Dark” Side of emotional intelligence are also revealed, which also may lead to the decline of EI effect sizes (Davis and Nichols, 2016). For example, some studies found that emotional attention may make influence of negative emotions bigger, which leads to depression in female. And emotional sensitivity may lead to stress reactivity, which is not conducive to interpersonal relationship (Davis and Nichols, 2016). What's worse, high emotional intelligence may lead to emotional manipulation and deception, which makes interpersonal relationship utilitarian and insincere (Grieve and Panebianco, 2013). In the early stage of the study, psychologists only focused on positive effects of emotional intelligence and ignored the negative effects of it, which may be the reason for overestimating effect sizes in mixed EI.

Therefore, how to improve the independence of mixed EI is an urgent problem. Only by reducing the overlap between mixed EI and other psychological structures will the prediction effects of it not be doubted. In follow-up studies, psychologists should reconsider the definition of mixed EI. And the most important, the attitude toward results should be changed, and scientists should not blindly pursue significant results, and ignore non-significant results. We should pay sufficient attention to the results that are non-significant or negative, and explain the positive results with caution. At present, many scientists advocate preregistration, that is, researchers openly register their studies before research (Miguel et al., 2014). Journals that have accepted a pre-registered study, will publish this study, regardless of its results significant or not, which makes it possible to publish non-significant results.

As for ability EI, this study has found no evidence that there are decline effects in ability EI research. And we consider that there are two possible explanations. One is that ability EI has a good independence, which will not be overestimated by the overlap with other psychological structures. The other explanation is that ability EI is measured by objective method, thus ability EI also has a good reliability and stability, there is little chance that effect sizes fluctuate over time.

This study also has an important finding that the average effect size of ability EI is significantly lower than that of mixed EI. The ability model is independent of other intellectual components, but also independent of the concept of personality. It does not include some psychological attributes, tendencies and characteristics of “non-ability” involved in mixed model, thus the predictive power of the ability model is relatively weak.

Although the present study shows that ability EI has good reliability, but this does not mean that it has a good validity, that is, effectiveness of measurement on emotional intelligence. The low predictive power of ability EI may also because its measurement of ability EI is not effective. This may be related to the definition of emotional intelligence in ability model. Ability model consists of four emotional ability: the ability to perceive, appraise, and express emotion; the ability to access and generate feelings which facilitate thought; the ability to understand emotion; and the ability to regulate emotions (Mayer et al., 1999). These four abilities cannot explain the complexity of emotional intelligence in interpersonal relationship. Therefore, the present study suggests that researchers in ability EI should reconsider the definition of emotional intelligence and expand its connotation in follow-up studies, in order to improve the effectiveness of its measurement. At the same time, studies in emotional intelligence should not be limited to ability model and mixed model, emotional intelligence should be expanded in more directions. At present, many studies begin to explore the evidence of emotional intelligence based on neuroscience (Waldman et al., 2011; Barbey et al., 2014; Boyatzis and Jack, 2018). Emotional intelligence is moving toward more scientific and objective approaches to research.

## Conclusion

We find evidences that there are decline effects in emotional intelligence field, and decline effects in the field of emotional intelligence are mainly due to the choice of emotional intelligence model and measurement method. Original studies in mixed EI may overestimate effect sizes, so effect sizes decrease obviously over time, whereas studies in ability EI have better stability and effect sizes in this field are not affected by time. However, the predictive power of the ability model is weaker than that of mixed model. Therefore, the problems to be solved in the field of mixed EI are how to reduce the overlap between mixed EI and other psychological structures, and how to improve the stability of the scale. As for ability EI field, how to improve the predictive power is a problem to be solved at present.

## Data Availability

The raw data supporting the conclusions of this manuscript will be made available by the authors, without undue reservation, to any qualified researcher.

## Author Contributions

ZG and XJ designed, performed, and analyzed the research. XJ wrote up the research. ZG critically reviewed and edited the manuscript.

### Conflict of Interest Statement

The authors declare that the research was conducted in the absence of any commercial or financial relationships that could be construed as a potential conflict of interest.
